# Salmonella meningitis in a young child from Pakistan: a case report

**DOI:** 10.1186/s13256-023-04128-0

**Published:** 2023-09-14

**Authors:** Imad Majeed, Sami Ullah Khan, Zaraq Rashid Khan, Sabawoon Hayat, Ihtisham Ullah, Asim Ali

**Affiliations:** 1 Lady Reading Hospital, Peshawar, Pakistan; 2 Khyber Teaching Hospital, Peshawar, Pakistan

**Keywords:** Salmonella, Meningitis, Infection, Communicable diseases, Infectious disease, Antibiotic resistance

## Abstract

**Background:**

Salmonella meningitis is a rare but serious complication of Salmonella infection, primarily affecting infants, children, and immunocompromised individuals.

**Case presentation:**

We present a case of a two-and-a-half-year-old Asian boy who developed Salmonella meningitis along with pneumonia and respiratory failure. Initially, he experienced symptoms of loose motions, fever, and irritability, which progressed to neck stiffness and brisk reflexes. Cerebrospinal fluid (CSF) analysis confirmed Salmonella typhi in the CSF. Due to the worsening condition, the patient was admitted to the intensive care unit, intubated, and switched to meropenem as the antibiotic of choice after an initial empiric therapy with ceftriaxone and vancomycin. With appropriate treatment, the patient showed significant improvement, including resolution of fever and respiratory symptoms.

**Conclusion:**

Management of Salmonella meningitis is often challenging primarily because of the fact that the empiric therapy for meningitis may not always provide coverage to the multi-drug resistant Salmonella species found in South Asia. Prompt administration of appropriate antibiotics based on sensitivity testing is crucial for successful management. This case emphasizes the importance of early recognition and effective management of this uncommon yet severe complication of Salmonella infection.

## Introduction

Salmonella Typhi, a Gram-negative bacillus, is the causative agent of typhoid fever, a systemic illness characterized by fever, headache, abdominal discomfort, and diarrhea [1]. Apart from the typical symptoms of typhoid fever, Salmonella Typhi can also lead to various extraintestinal complications, including osteomyelitis, endocarditis, hepatitis, cholecystitis, and meningitis, which necessitate timely diagnosis and treatment. [2]. It has been estimated that globally, around 35 million cases of Salmonella Typhi infection occur every year, resulting in approximately 500,000 deaths [3]. Salmonella meningitis is an infrequent yet severe complication of Salmonella infection, predominantly affecting infants, children, and immunocompromised individuals [4, 5]. While Salmonella meningitis is a rare disease in developed countries, accounting for only 1% of cases, it poses a significant health concern in developing nations, where it affects a considerably higher proportion of infants and children, around 13% [6]. The mortality rate of this condition remains alarmingly high at approximately 90%, regardless of the serotype involved [6]. Salmonella meningitis can manifest with non-specific symptoms, including fever, irritability, lethargy, and altered mental status, making its diagnosis challenging. Isolation of the organism from either cerebrospinal fluid (CSF) or blood cultures is essential for the confirmation of this condition [7]. Management of Salmonella meningitis remains a complex and non-standardized process. Over the past few decades, numerous drugs have been employed in treating this condition, including chloramphenicol, ampicillin, co-trimoxazole, third generation cephalosporins, and fluoroquinolones [8]. The presence of serious medical illness precipitated by unhygienic food practices can worsen the prognosis of this serious infection [9]. In this report, we present a case of a two-and-a-half-year-old male patient who developed Salmonella meningitis complicated by pneumonia and respiratory failure following a gastrointestinal infection. We also review the literature on the epidemiology, pathogenesis, clinical features, diagnosis, treatment, and prevention of Salmonella meningitis.

## Case presentation

A two-and-a-half-year-old Asian male patient presented to the emergency department with the chief complaints of loose bowel motion, fever, and irritability for the last 5 days. According to his father, the loose bowel motions were sudden in onset and watery in nature. He had a total of 7–8 daily episodes and was associated with abdominal pain. The patient subsequently developed a high-grade fever of 101F that was intermittent in nature and relieved by paracetamol. He also progressively got irritable during this time frame.

On arrival, he was lethargic and drowsy, with a Glasgow Coma Scale (GCS) score of 9. His blood pressure was 100/60 mmHg and his pulse was 103 bpm. He had normal vesicular breathing on chest auscultation, and both the abdomen and cardiovascular examination was unremarkable. His neurological assessment revealed a positive Brudzinski’s sign and generalized brisk deep tendon reflexes graded at 3+. He also had a positive Babinski’s reflex.

**Fig. 1:**
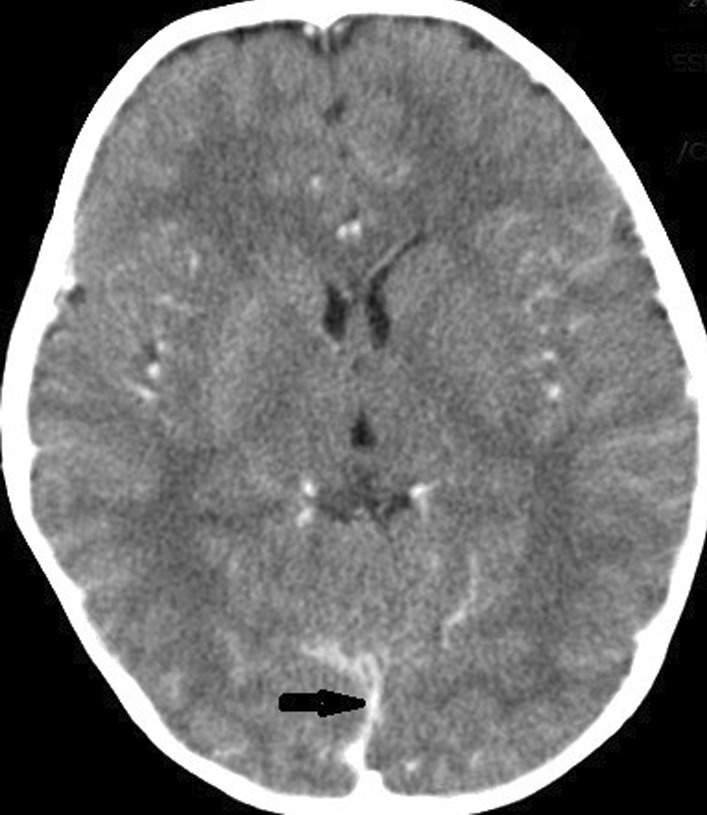
A computed tomography scan of brain shows generalized meningeal enhancement (The arrow points towards meningeal enhancement)

On the 1st day of admission, baseline investigations, blood culture sensitivity and CSF studies including routine analysis and culture sensitivity were advised. A contrast enhanced Computed Tomography scan (CT scan) of the brain was also done prior to CSF studies. He was then started on empirical intravenous antibiotics including ceftriaxone 75 mg/kg/day in divided dose and vancomycin 20 mg/kg every 6 hourly. He was also started on infusion ringer lactate 120 ml/kg/day, and injection dexamethasone 0.6 mg/kg/day. His vitals were constantly monitored via a cardiac monitor. His blood work revealed a white blood cell count of 21.1 × 10.e3/μl comprising 90% neutrophils and 8% lymphocytes, and a C-reactive protein level greater than 30.4 mg/dl. Rest of the baseline metabolic panel was within the normal range, including serum urea, creatinine, alanine transaminase, serum electrolytes, calcium, magnesium, and serum albumin. His CSF regular examination report revealed a protein level of 374 mg/dl, a glucose level of 4 mg/dl, and a white blood cell count of 1840/cmm comprising of 96% neutrophils and 10% lymphocytes. Gram-negative rods were observed in the gram staining of the CSF regular examination. His CT scan revealed generalized meningeal enhancement as shown below (Fig. 1).

**Fig. 2:**
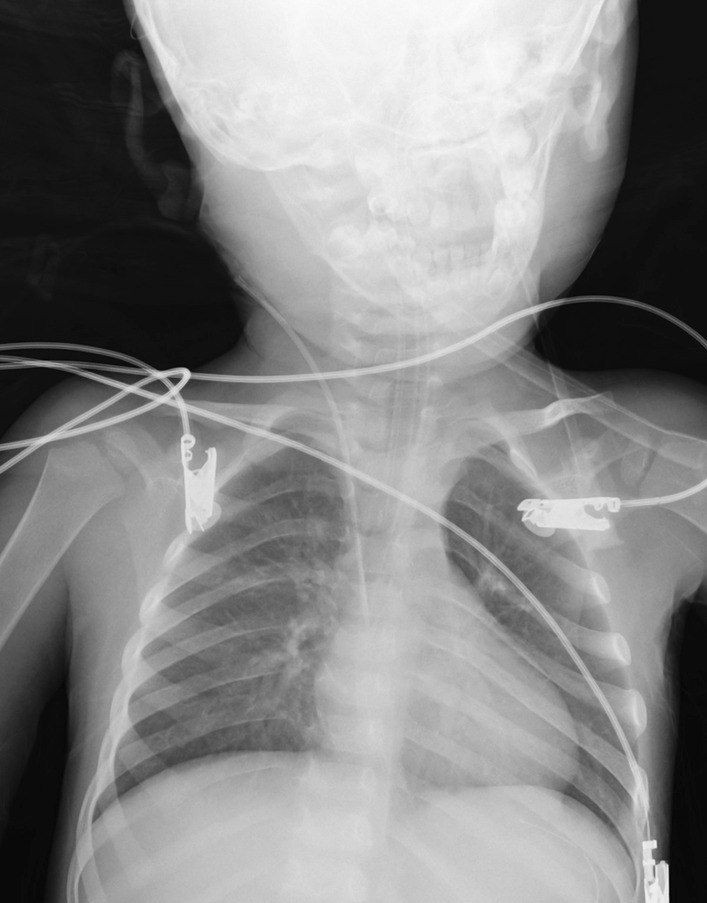
A chest X-ray shows bilateral upper zone opacities and right hilar shadow

The patient’s condition deteriorated the following day as he developed tachypnea with a respiratory rate of 40. His oxygen saturation on pulse oximetry was recorded at 88% on room air. He had right sided crackles upon chest auscultation. Therefore, he was started on 5 L of supplemental oxygenation and a chest X-ray was advised which revealed bilateral upper zone opacities and right hilar prominence (Fig. 2). His arterial blood gas analysis revealed respiratory alkalosis with a pH of 7.494, pCO_2_ of 31.5 mmHg, pO_2_ of 79.6 mmHg, and HCO_3_ levels at 23.7 mmol/l.

On the 3rd day of admission, his condition deteriorated. Therefore, he was transferred to the intensive care unit, where he was intubated, and a specimen was taken from his tracheal aspirate that was sent for culture and sensitivity. The patient’s preliminary CSF culture report came after 48 hours that showed heavy growth of Salmonella typhi, which was sensitive only to imipenem and meropenem. Therefore, his antibiotics were switched to meropenem at dosage of 40 mg/kg IV every 8 hours. Dexamethasone was also stopped at this point.

On the 5th day of admission, his tracheal aspirate culture and sensitivity report came that was positive for *Klebsiella pneumonia* and was sensitive to antibiotics including meropenem. He had developed the pneumonia in the setting of aspiration due to his altered mental status. While his blood culture report came back negative. The patient at this point showed significant clinical response to the treatment both in terms of fever spikes and amount of oxygen required. A repeat blood work-up was advised which revealed white blood cell count levels of 13.1 × 10.e3/μl and a C-reactive protein (CRP) level of 15.8 mg/dl.

The patient was extubated on the 7th day of admission. He was shifted back to ward floor. His blood pressure was 110/75 mmHg and his pulse was 70 bpm. His respiratory rate was recorded at 25/min. His fever had subsided at this point. He still had mild crackles on chest examination. He had a GCS of 15/15 and had started taking normal feed. Therefore, on the 8th day of admission, he was discharged on home medications including meropenem which was to be administered through a PICC line at 40 mg/kg every 8 hourly for a total duration of 28 days and paracetamol syrup as needed. Although, the duration of intravenous meropenam for 28 days predisposed the patient to significant side effects but the decision was taken by the our clinical team after assessing risks versus benefits of the drug. The patient presented for a follow-up visit to our outpatient department after 14 days and was found to have completely recovered.

## Discussion

Meningitis is inflammation of the meninges resulting in swelling of membranes [10]. A bacterial or viral infection usually accounts for the cause of meningitis. However, injuries, cancer, certain drugs and other types of infections can also cause meningitis [11]. It is important to determine the specific bug causing meningitis because this eventually dictates the treatment options [12].

Salmonella Typhi is a Gram-negative bug that commonly affects the intestinal tract, resulting in “Enteric fever” [13]. The usual clinical manifestations of Salmonella infection include gastroenteritis, enteric fever, bacteremia, and asymptomatic carriage. However, a rare clinical presentation and complication of Salmonellosis is Salmonella meningitis [14].

Salmonella meningitis is not widely reported in the literature and only a small number of cases have been reported to date. A study reported in Ibadan showed Salmonella typhi meningitis in an 11-month-old infant who presented with a short history of loose motions and seizures [15]. A case series of two infants by Owuso-Ofori *et al.* in July 2000 reported severe mortality by this disease if not promptly treated within the first 48 hours [16]. A case series conducted in Georgia showed the occurrence of Salmonella meningitis in HIV patients. This study also concluded that along with other opportunistic infections, Salmonella meningitis has a 70% more chance of occurring in an immuno-compromised individual [17].

Symptoms of Salmonella meningitis are similar to other forms of meningitis, commencing suddenly and escalating acutely. Various symptoms include fever, seizures, severe headache (95%), neck stiffness (44%), and neurological deficits (33%) [18]. A case series done in Taiwan reported the occurrence of seizures in 63% of Salmonella meningitis patients before hospitalization and in 54% of patients during hospitalization pointing to seizures being one of the common symptoms of the disease [19]. A retrospective study done in Malaysia reported 13 infants with Salmonella meningitis. Out of those 13, nine developed fits, five had hydrocephalous, four had subdural effusions and three had empyema [20]. Our case comprised a two and half-year-old male patient who presented with complaints of loose motions, fever and irritability.

Blood culture sensitivity, cerebrospinal fluid (CSF) culture sensitivity and CSF routine analysis are commonly used for the diagnosis of Salmonella meningitis [21]. A case report in South Africa revealed increased polymorphonuclear leukocytes, excess globulins, and proteins above 500 mg/dl in CSF of Salmonella meningitis patients [22]. A case reported by Singhal *et al.* elaborated Salmonella meningitis in a 28-day old infant, whose blood and CSF culture showed Salmonella typhi growth. CSF analysis in that patient revealed 927 mg/dl of protein, 1 mg/dl of glucose and 2050 leukocytes per mm^3^ (N-40%, L-52%, E-2%, M-6%) [23]. A rare case reported in BMC infectious disease revealed high protein (9.9 g/l) and decreased glucose (0.1 mmol/l) in CSF of a Salmonella meningitis patient along with increased serum erythrocyte sedimentation rate (125 mm/h) and increased C-reactive protein level (130 mg/dl) pointing to that fact that high inflammatory markers can also be non-specific supporting evidence in Salmonella meningitis patients [24]. In our case, the CSF report revealed a total leukocyte count of 1840/mm^3^ including 96% neutrophils and 10% lymphocytes, a protein level of 374 mg/dl and a glucose level of 4 mg/dl. Our patient also had a high C-reactive protein level of 30.4 mg/dl.

Treatment of Salmonella meningitis is more or less the same as treating any manifestation of salmonellosis; however, Salmonella species have a wide range of antimicrobial resistance [25]. Antimicrobial susceptibility of different isolates of Salmonella was assayed from September 2003 to February 2004 which revealed 32.7% of Salmonella isolates were resistant to one or more of 24 antimicrobials tested. The most common resistance was to streptomycin (75%), ampicillin (59.4%), tetracycline (46.9%), and sulfisoxazole (40.6%). Around 83.3% of isolates showed multidrug resistance [26]. A study conducted by Owuso *et al.* reported that fluoroquinolones had a cure rate of 88.9%, while the third-generation cephalosporin had a cure rate of 84.6% [16]. In children the rate of Salmonella clearance in CSF was more rapidly achieved with imipenem or ceftriaxone than with co-trimoxazole or chloramphenicol [8]. In our case the CSF culture showed sensitivity to imipenem and meropenem only. Patient was started on meropenem and achieved dramatic clinical response.

## Conclusion

The aim of this case report is to highlight the importance of rare manifestations of Salmonella, especially considering the fact that the prevalence of Salmonella infections is increasing in developing countries. This case reports also highlights the fact that we as a medical community face a daunting task of dealing with highly resistant bugs, so more awareness regarding antibiotics stewardship and the need for developing new antibiotics is required.

## Data Availability

Not applicable.

## References

[1] Rahman T, Hosen I, Chakraborty S (2013). A rapid glimpse on typhoid fever: an updated mini review. J Life Med.

[2] Khan KH, Ganjewala D, Rao KB (2008). Recent advancement in typhoid research—a review. Adv Biotech.

[3] Nwadike VU, Fowotade A, Tuta KE, Olusanya OO (2012). A rare case of *Salmonella* typhi meningitis in an eleven month old infant: a case report. Ann Ib Postgrad Med.

[4] Al-Aani FK, Abusalah S, Al-Aqeedi R, Ibrahim A (2009). *Salmonella* meningitis in an adult with type B viral hepatitis and an incidental schwannoma. BMJ Case Rep..

[5] Carr BG, Weisbein JL, Gaieski DF (2011). *Salmonella* meningitis in an immunocompetent adult. J Emerg Med.

[6] Khurshid N, Khan BA, Bukhari SW, Shahid A, Punshi A (2019). Extensively drug-resistant *Salmonella* typhi meningitis in a 16-year-old male. Cureus.

[7] Mahalakshmi R, Rajeshbabu B, Mohan R, Balakumaran D, Venkataraman P, Ponnurangam NV (2013). *Salmonella* paratyphi B meningitis in an infant. Australas Med J.

[8] Price EH, de Louvois J, Workman MR (2000). Antibiotics for *Salmonella* meningitis in children. J Antimicrob Chemother.

[9] Lim SY, Lee KW, Seow WL, Mohamed NA, Devaraj NK, Amin-Nordin S (2021). Effectiveness of integrated technology apps for supporting healthy food purchasing and consumption: a systematic review. Foods.

[10] Feigin RD, Mccracken GH, Klein JO (1992). Diagnosis and management of meningitis. Pediatr Infect Dis J.

[11] Bartt R (2012). Acute bacterial and viral meningitis. Infect Dis.

[12] Kaplan SL, Feigin RD (1983). Treatment of meningitis in children. Pediatr Clin N Am.

[13] Ispahani P, Slack R (2000). Enteric fever and other extraintestinal salmonellosis in University Hospital, Nottingham, UK, between 1980 and 1997. EJCMID.

[14] Goldberg MB, Rubin RH. The spectrum of *Salmonella* infection. Infect Dis Clin N Am. 1988;2(3):571–598. ISSN 0891-5520. 3074116

[15] Nwadike VU, Fowotade A, Tuta KE, Olusanya OO (2012). A rare case of *Salmonella* typhi meningitis in an eleven month old infant: a case report. Ann Ib Postgrad Med.

[16] Owusu-Ofori A, Scheld WM (2003). Treatment of *Salmonella* meningitis: two case reports and a review of the literature. Int J Infect Dis.

[17] Leonard MK, Murrow JR, Jurado R, Gaynes R (2002). *Salmonella* meningitis in adults infected with HIV: case report and review of the literature. Am J Med Sci.

[18] Griffiths M, McGill F, Solomon T (2018). Management of acute meningitis. Clin Med.

[19] Wu HM, Huang WY, Lee ML (2011). Clinical features, acute complications, and outcome of *Salmonella* meningitis in children under one year of age in Taiwan. BMC Infect Dis.

[20] Lee WS, Puthucheary SD, Omar A (1999). *Salmonella* meningitis and its complications in infants. J Paediatr Child Health.

[21] Monica F, Valentina C, Lisa M, Beatrice R, Simone F, Greta C, Barbara P, Alberto B, Laura L, Lorenzo I (2019). Unusual meningitis caused by non-typhoid *Salmonella* in an Italian infant: a case report. Acta Biomed.

[22] Watson KC (1958). *Salmonella* meningitis. Arch Dis Child.

[23] Singhal V, Saleem EK, Rajesh SM, Coutinho A (2012). Neonatal *Salmonella* typhi meningitis: a rare entity. J Clin Diagn Res.

[24] Rule R, Mbelle N, Sekyere JO, Kock M, Hoosen A, Said M (2019). A rare case of colistin-resistant *Salmonella* enteritidis meningitis in an HIV-seropositive patient. BMC Infect Dis.

[25] Erdem B, Ercis S, Hascelik G, Gur DENİZ, Gedikoglu S, Aysev AD, Sumerkan B, Tatman-Otkun M, Tuncer I (2005). Antimicrobial resistance patterns and serotype distribution among *Salmonella enterica* strains in Turkey, 2000–2002. Eur J Clin Microbiol Infect Dis.

[26] Zewdu E, Cornelius P (2009). Antimicrobial resistance pattern of *Salmonella* serotypes isolated from food items and personnel in Addis Ababa, Ethiopia. Trop Anim Health Prod.

